# A New Paradigm for Safety Data Signal Detection and Evaluation Using Open-Source Software Created by an Interdisciplinary Working Group

**DOI:** 10.1007/s43441-021-00319-3

**Published:** 2021-07-19

**Authors:** James Buchanan, Mengchun Li, Xiao Ni, Jeremy Wildfire

**Affiliations:** 1 Covilance, LLC, 2723 Sequoia Way, Belmont, CA 94002 USA; 2 TB Alliance, New York, NY USA; 3 Sarepta, Inc., Boston, MA USA; 4 Gilead Sciences, Foster City, CA USA

**Keywords:** Drug safety, Pharmacovigilance, Interactive graphics

## Abstract

Techniques to evaluate large amounts of safety data continue to evolve based on a greater understanding of how the brain processes visual information and the advancement of programing tools. The Interactive Safety Graphics Task Force of the American Statistical Association Biopharmaceutical Safety Working Group has assembled a multidisciplinary team of experts in a variety of domains to develop the next generation of open-source visual analytical tools for safety data based on these advances. The multidisciplinary approach resulted in the rapid development of the first tool, a novel interactive version of the familiar Evaluation of Drug-Induced Serious Hepatotoxicity (eDISH) graphic along with a unique clinical workflow to guide the reviewer through the data analysis. This now serves as the model for the team to expand the open-source platform into a suite of other interactive safety analysis tools.

## Background

Safety monitoring during clinical trials is an essential component in drug development. Thorough reviews of medical safety data at regular intervals are critical to characterize the drug safety profile as early as possible to protect patient safety and, eventually, public health. Traditionally, safety data were only comprehensively reviewed at the end of trials. Safety data from ongoing studies, when available, are typically presented in long tedious listings, which are time-consuming to review and less intuitive to inform critical insights. Hence, a thorough review is difficult to conduct on an ongoing basis. As analytical tools became available, comprehensive safety data could be reviewed in using static graphics, usually at certain planned time points. While an improvement on the less informative listings, static graphics are still of limited utility since they do not allow patient-level data exploration, nor population-level ad hoc analyses related to questions arising during the review process. With these inefficient methods, safety data reviews during clinical trials are less frequent and less comprehensive than they ideally should be performed. The result is that safety signals are not identified promptly, and the evaluation of these signals is delayed leading to unnecessary risk in the study patient population. Obviously, this is not in the best interest of any of the various stakeholders during clinical development.

An interactive graphical tool would facilitate ongoing, timely, and flexible safety data exploration to identify safety signals as well as offer capabilities to evaluate events of interest at a population level and the cases of interest at a patient level. Yet, interactive safety displays also have limitations; many such tools do not guide the user as to how to best utilize their features to resolve the important clinical questions when evaluating a safety signal. Graphical display tools are most powerful when paired with an appropriate medical approach to interrogate the data for evidence for or against a causal association between the safety finding and the study drug. Thus, the development of a medically valid clinical workflow with suggested evaluations and guidance as to their interpretation greatly improves the utility of the interactive tool, while also encouraging collaboration between the clinician and statistician to perform more in-depth analyses that are prompted by the data review. The construction of such an interactive tool and matching clinical workflow is clearly a task that no single statistician, programer, or clinician can fulfill. Interdisciplinary teamwork is required to bridge the realms of medical science and data science.

In 2017, a group of statisticians from the American Statistician Association (ASA) were attempting to develop interactive graphical tools but faced a bottleneck as they needed guidance on what were the right clinical safety questions to address, and what were the best ways to explore the data to resolve these questions. At the same time, a group of clinicians from the Drug Information Association (DIA) were looking for experts who could help them transforming long, tedious data listings into useful information to better address their safety questions. Serendipitously, these two groups met, realized their common goals, and quickly came up with a solution—a multidisciplinary working group. Thus, the ASA–DIA joint safety working group was formed. A specialized task force within the working group, the interactive safety graphics (ISG) task force, is dedicated to the development of open-source, novel, interactive safety graphics tools to assist with the evaluation of safety risks of investigational drugs.

FDA’s Premarket Risk Assessment guidance [1] identifies areas that each sponsor should bear in mind when evaluating their accumulating safety data. The ISG task force decided that the interactive safety graphic tool should focus first on these areas of most relevance to safety evaluations. As a result, the ISG task force started with hepatotoxicity, which is arguably the principal safety topic of concern for any given compound in development. Built based on the concept of Hy’s law [2] and the eDISH tool developed by the US FDA [3], the ISG task force envisioned that the open-source interactive analytic tool should draw on emerging analytical science and new approaches to explore the data for instances of possible drug-induced liver injury (DILI), and in a manner that would be broadly applicable to the pharmaceutical industry, academic institutions, data monitoring committees, and regulatory agencies.

Collaborations with other world-class groups, such as the Council for International Organizations of Medical Sciences (CIOMS) Drug-Induced Liver Injury (DILI) Working Group, Pharmaceutical Users Software Exchange (PhUSE), and expert hepatologists helped to realize the goal. The extent of both internal and external collaborations is indeed a paradigm shift for creating tools to assist in signal detection and safety evaluation in drug development.

## Team Formation and Project Objectives

To facilitate the development of a tool that is in line with the vision, it was recognized at the outset that the team must be multidisciplinary with expertise in clinical science, data science, programing, and principles of graphical data display.

From a regulatory environment point of view, the timing is in favor of such an initiative. In 2010, US FDA published a draft rule amending the investigational new drug (IND) safety reporting requirement [4]. In December 2012, US FDA issued its final guidance for industry and investigators on safety reporting requirements for IND and bioavailability/bioequivalence (BA/BE) studies [5]. The guidance provided the agency’s current thinking on its expectation for timely review, evaluation, and submission of relevant IND safety information from clinical trials and other activities under the IND regulations. In 2015, the FDA issued draft guidance on safety assessment for IND safety reporting which provided advice to sponsors on developing a systematic approach for aggregate safety assessment for human drugs and biological products developed under an IND [6]. This favorable regulatory environment and the clear gaps in the existing toolset for safety monitoring allowed the founding team members of our team to quickly assemble a cross-functional team that includes leading statisticians from ASA, a seasoned safety signal detection instructor from DIA, experienced safety physicians from the industry and FDA, and outstanding programers and data scientists who specialize in safety graphics.

Group membership is open and dynamic and, as such, the team has expanded over the course of the project. It was helpful that the leadership maintained a balance between the predefined goals and a flexible growth mindset. As more people join a team, it is easy to get distracted or drift from the primary purpose if there is no clear goal. On the other hand, new ideas and novel approaches may emerge during the course of development that offers improved features, more efficient code, or other enhancements to the tool when new people join the effort. When empowered properly, new ideas enhance the final product rather than distract from it.

The concept of interactive graphic tools is nothing new to statisticians and programers; however, its uptake with the end-users, safety reviewers, has been limited, perhaps due to the lack of communication between these two groups. Many previous tools were developed without sufficient input from end-users: features were not intuitive to use, or the tool did not adequately address the question posed by the safety reviewer. The ISG task force learned from these lessons and decided to approach the development of the tool with a close collaboration between clinicians, data scientists, and statisticians from day one. Development was an iterative process with each display graphic, analytical feature, or data handling method reviewed by the interdisciplinary team. This process is consistent with the concept of “design thinking” which is a creative tool to form action-oriented ideas using the varied perspectives of a cross-functional team [7].

The variety of interactive features was also intended to leverage the safety reviewer’s natural intelligence into a state of augmented intelligence, a step along the way to developing an artificial intelligence model. This speaks to the concept of “regulatory centaurs,” a combination of human and machine intelligence that augments the key features of each in a manner that can improve pharmacovigilance [8].

When the ISG task force started to plan for the specific objectives, the multidisciplinary team members came to the consensus that the interactive safety graphic tool should be as follows:

- *Open-source* Commercially designed interactive visualization tools are available; however, the cost of these proprietary tools may be a barrier to their broad adoption. Additionally, regulatory authorities cannot be placed in a position where they appear to support a particular commercial product. The ISG task force aimed to develop a tool that is free to all interested users.
- *Easy to use web-based application* This is particularly important for the non-technically experienced safety reviewer in a resource-constrained setting. Any technical hurdle that stands in the way may further prevent a clinician from using the tool. An easily operated web-based interactive application will minimize the technical requirements for its use.
- *Data format agnostic* Although the Clinical Data Interchange Standards Consortium’s (CDISC) Analysis Data Model (ADaM) provides a useful standard during clinical development, a CDISC-compliant dataset may only be available at certain intervals and not on a real-time basis that follows the frequency of ongoing data analysis. The ISG task force realized that the tool’s utility improves when it supports non-standard data formats. As such, our tools automatically recognize ADaM or SDTM datasets, but if the data format is not standardized then a simple data mapping can be performed by the user.
- *Easily configurable* The application should be easy to use on its own but should also be capable of incorporation into existing sponsor systems. The application is built in JavaScript and R allowing the end-user to easily configure it for their own needs.
- *Encourages collaboration between clinicians and statisticians* As described earlier, the task force decided to take an end-to-end approach when developing the tools. The collaboration between clinicians, data scientists, and statisticians has produced a tool that addresses the actual needs of the safety reviewer. As the tool is designed for data exploration, the safety reviewer is anticipated to identify additional areas of inquiry that are best served by a collaboration with their statistician and programer colleagues.
- *Broadly applicable to industry, regulatory authorities, data monitoring committees (DMCs), and academic communities* These entities share the common goal of understanding the safety profile of a drug. All have resource constraints that impact the timely and efficient review of safety data. An additional challenge faced by organizations is how to minimize inter-reviewer variability in the interpretation of the data and the conclusions that they draw. A standardized methodology to review and evaluate safety data, as provided by the accompanying clinical workflow, improves the efficiency of an organization and also facilitates better communication between entities. Just as CDISC became a standard data format applicable to industry and regulatory authorities, a standardized approach to safety data analysis may also streamline data review and provide a common platform for sharing data and analyses between industry and regulatory authorities.

## Interactive Safety Graphic: Hepatic Explorer

### Overview

With these goals in mind, the ISG released the Hepatic Explorer (https://safetygraphics.github.io/hep-explorer/) in early 2019. The Hepatic Explorer is an interactive graphic for exploring hepatic laboratory data from clinical trials based on the well-established eDISH tool [3].

By embracing the ISG principles outlined above, the Hepatic Explorer builds upon the static eDISH graphic in several important ways. First and foremost, the tool is completely free to use, open-source, and easy to share. This allows for easy implementation for both internal and external use cases, which can be difficult for both commercial tools and company-specific eDISH implementations. The tool itself has all the standard features found in existing interactive eDISH graphics. The robust collaboration between technical and clinical team members has also facilitated the implementation of new features that are not commonly available, including the following:

- Click a point to generate an associated participant profile.
- Gap Minder-style animation of lab values over time (www.gapminder.org). The animated hysteresis plot depicts how the lab values change relative to each other over time.
- Multiple grouping and filtering options based on data available in the dataset.
- Ability to modify the *X* and *Y* axes to display fold change from baseline rather than fold change from the upper limit of normal.
- Change the *X*-axis to display the nR value to use the nR modified Hy’s Law approach [9].
- Automatic calculation of *P*_ALT_, an estimate of the extent of hepatocyte loss associated with an elevation of alanine aminotransferase (ALT) [10]
- Ability to filter and plot the R Ratio and nR Ratio, a measure of the extent to which the data reflect a hepatocellular, cholestatic, or mixed process [9, 11, 12]
- Dynamic axes and threshold points to accommodate situations warranting a modification of ALT and bilirubin cut points, e.g., oncology studies [13]
- Adjustment of point size based on various measures, e.g., alkaline phosphatase value, R ratio.
- Color coding points based on time separation of peak measures. The time range is user-defined.
- Drill down to patient-level data to display how key analytes change over time, and how they change relative to the calculated R Ratio value. From these spaghetti plots, how these analytes increase and decrease over time provides insight into possible mechanisms of toxicity.
- Easy export of the graphic in a fully self-contained, interactive html report.

### Clinical Workflow

**Figure 1:**
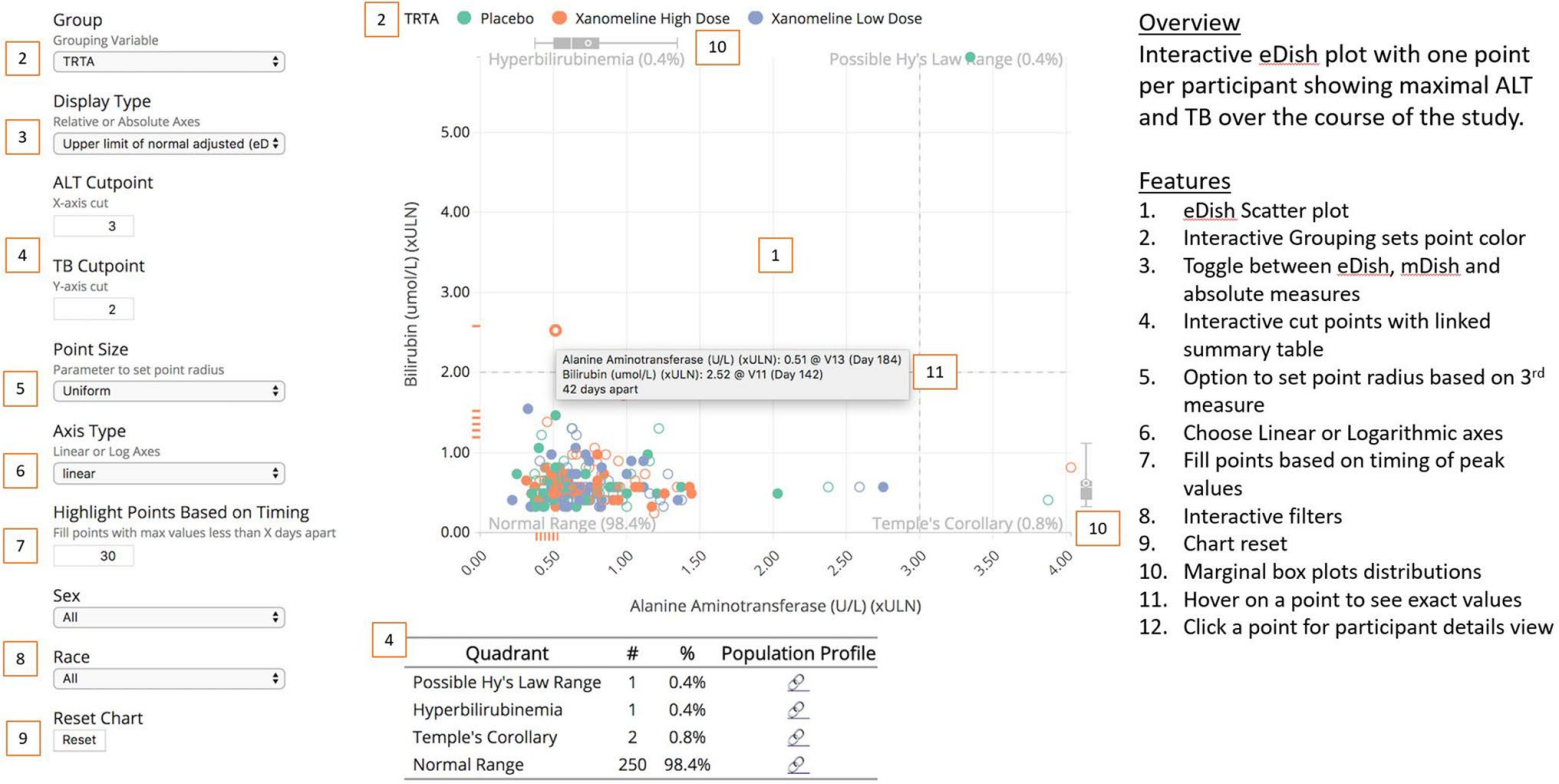
Hepatic Explorer Features.

A crucial and unique component of the Hepatic Explorer is a robust clinical workflow (https://github.com/SafetyGraphics/SafetyGraphics.github.io/raw/master/guide/HepExplorerWorkflow_v1_2.pdf) that describes in great detail a series of analyses to perform with the tool. For each of the quadrants of interest—possible Hy’s Law, Temple’s Corollary, and hyperbilirubinemia—the reviewer is guided through a sequence of steps. Each is accompanied by a textual discussion of the purpose of the analysis and how to interpret the results. Each is referenced to the supporting medical literature. At the end of the process, the reviewer will have accumulated information that supports or discounts a possible causal role for the drug. A summary version of the workflow is also available to facilitate rapid triage of cases by safety reviewers to a company hepatic review board. As the state of the art evolves, the clinical workflow is updated to capture cutting-edge knowledge (Fig. 1).

### Technical Details

The Hepatic Explorer is written in javascript using webcharts [14] and D3.js [15] and uses a technical framework that is closely related to the tools introduced as part of the Safety Explorer Suite [16]. The Hepatic Explorer can be run in any modern web browser and on a variety of platforms. It supports all common laboratory data standards and can also be run on non-standard laboratory data after a simple data mapping process. Users can create an instance of the Hepatic Explorer as a standalone webpage, but our team has also developed the safetyGraphics R package (https://cran.r-project.org/package=safetyGraphics) to allow users to initialize the chart from any set of laboratory results using a simple graphical user interface. The Hepatic Explorer has undergone extensive testing before release and subsequently has been used in contract research organizations, academic institutions, and in both large and small pharmaceutical companies.

Data security in such an application is, of course, a potential concern. As with other tools, the users and their IT group need to enable importing or linking to the source data securely and are ultimately responsible for the security of their data. In most organizations, there should be such SOPs or guidance from IT and quality assurance for accessing confidential data. As part of our Version 2.0 release, the team is developing a vignette on this specific topic with the purpose to inform users of potential data security risks and guide them to a secure deployment. The data safety procedures and considerations depend on the method of deployment. For example, if the users run the application and import the datasets locally, then the data can be secured within their organization’s firewall. On the other hand, we advise against deploying to a public non-secure server, such as shinyapps.io, if the desire is to upload private data there for analysis.

## Future Developments and Uptake

The task force has developed a road map (https://safetygraphics.github.io/roadmap) to articulate plans for improvements to existing graphical tools as well as new analytical tools. Based on the areas of interest described in the FDA’s Premarket Risk Assessment guidance [1], the task force plans to also develop tools that address renal toxicity and QTc prolongation. In keeping with the Hepatic Explorer, these will offer interactive features and be paired with a clinical workflow. Concerning renal toxicity, work is underway to create a Renal Explorer that will identify instances of acute kidney injury based on the Kidney Disease: Improving Global Outcomes (KDIGO) [17], Risk, Injury, Failure, Loss of kidney function, and End-stage kidney disease (RIFLE) [18] and Delta Creatinine criteria [19]. The QT prolongation tool will explore the use of new electrophysiological biomarkers.

One benefit of the open-source platform and the multidisciplinary nature of the team has been that programing efforts at other companies have been voluntarily offered for inclusion in the safety graphics platform. The interactive volcano plot is one example of a tool initially developed outside of the task force but was graciously provided by that sponsor for the team to build upon. This exemplifies how our collective efforts can expand the utility of the tool suite to the benefit of everyone. A shift from each company building their own tools to a shared development environment is a more efficient use of everyone’s resources.

Pharmaceutical companies have repeatedly endeavored to be allowed to submit packages for regulatory review using their proprietary data review systems. Understandably, the regulatory agencies have balked at the prospect of having to be trained on a multitude of such proprietary systems. However, a common analytical framework that is open-source could be a path to solve this dilemma. The task force is in discussions with organizations like PhUSE to explore the possibility of including the Hepatic Explorer in submissions to the FDA.

Of course, the utility of a tool is only as great as the knowledge of its availability and capabilities. Team members of the task force have reached out to the academic community, US FDA, and hepatology groups such as the Council for International Organizations of Medical Sciences (CIOMS) DILI Working Group, and the IQ Consortium Drug-Induced Liver Injury Initiative (IQ-DILI). The deliverables have also been presented at various professional meetings including those for the pharmaceutical industry (e.g., DIA), hepatology conferences (e.g., AASLD), statistical conferences (e.g., JSM), and technical conferences (e.g., RStudio conference, R in Pharma). The scope of the audience mirrors the range of expertise in our multidisciplinary team and the goal of informing all relevant stakeholders of the details of this tool. Communication to various professional organizations is supplemented by individual presentations to a variety of pharmaceutical companies and targeted training sessions. As a result of these extensive outreach efforts, the tool had been downloaded from CRAN (https://cran.r-project.org/package=safetyGraphics) on an RStudio mirror site 9820 times as of April 2021. One benefit of reaching out to multiple organizations is finding like-minded individuals who are interested in participating in further developing this open-source tool suite. This further strengthens the collaborative multidisciplinary nature of the team.

## Conclusion

What started as the recognition of joint interest between safety scientists, statisticians, and data scientists has developed into a multidisciplinary effort that has eclipsed the initial vision. The multidisciplinary team has shown the capacity to rapidly develop novel safety graphics that better address the needs of safety reviewers in an environment that increasingly recognizes the value of regular ongoing data review. The accompanying clinical workflow allows reviewers to conduct a more thorough clinical evaluation. The open-source nature makes the tools more broadly available than locally developed or proprietary solutions. Although initially developed with the needs of the pharmaceutical industry in mind, the interest expressed among academic, regulatory, and DMC entities has been gratifying [20]. The possibility that a tool suite with broad analytical capabilities that is readily accessible to all raises the possibility that the platform might represent a new standard that could facilitate communication with regulatory authorities. Finally, participation from multiple pharmaceutical companies has shown that by pooling expertise and resources such novel tools can be developed more quickly and efficiently in a collaborative, pre-competitive environment than the prior model of each company working independently and redundantly.
